# Untangling the role of RhoA in the heart: protective effect and mechanism

**DOI:** 10.1038/s41419-024-06928-8

**Published:** 2024-08-09

**Authors:** Shigeki Miyamoto

**Affiliations:** Department of Pharmacology, University of California, San Diego, La Jolla, CA 92093-0636 USA

**Keywords:** Experimental models of disease, Cardiovascular biology

## Abstract

RhoA (ras homolog family member A) is a small G-protein that transduces intracellular signaling to regulate a broad range of cellular functions such as cell growth, proliferation, migration, and survival. RhoA serves as a proximal downstream effector of numerous G protein-coupled receptors (GPCRs) and is also responsive to various stresses in the heart. Upon its activation, RhoA engages multiple downstream signaling pathways. Rho-associated coiled-coil-containing protein kinase (ROCK) is the first discovered and best characterized effector or RhoA, playing a major role in cytoskeletal arrangement. Many other RhoA effectors have been identified, including myocardin-related transcription factor A (MRTF-A), Yes-associated Protein (YAP) and phospholipase Cε (PLCε) to regulate transcriptional and post-transcriptional processes. The role of RhoA signaling in the heart has been increasingly studied in last decades. It was initially suggested that RhoA signaling pathway is maladaptive in the heart, but more recent studies using cardiac-specific expression or deletion of RhoA have revealed that RhoA activation provides cardioprotection against stress through various mechanisms including the novel role of RhoA in mitochondrial quality control. This review summarizes recent advances in understanding the role of RhoA in the heart and its signaling pathways to prevent progression of heart disease.

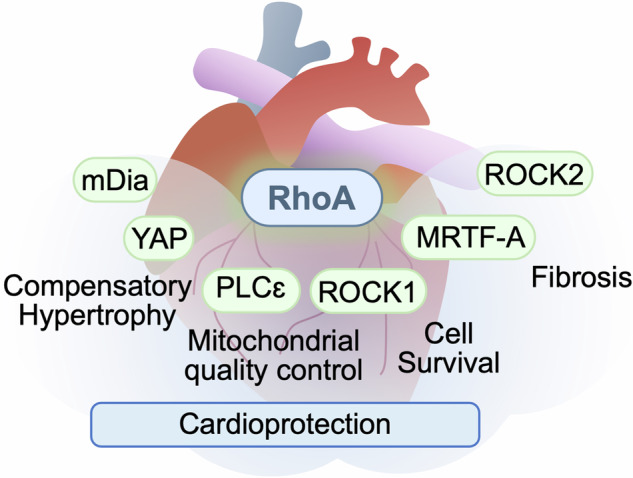

## Facts

- RhoA functions as a signaling hub that senses a variety of upstream signals such as GPCRs, mechanical force and oxidative stress.
- RhoA engages various downstream effectors such as ROCK, YAP, MRTF-A and PLCε to regulate cardiac pathophysiology.
- Activation of RhoA is not inherently pathological but provides protection in the heart against stress, such as pressure-overload, ischemia and aging, but induces fibrosis.
- Mitochondrion is a critical point of convergence for RhoA-mediated cardioprotection against ischemic stress.

## Open questions

- Does RhoA regulate other mitochondrial quality control mechanisms besides PINK1/Parkin mitophagy to provide cardioprotection?
- Is general autophagy also regulated by RhoA in the heart?
- How does RhoA signaling pathway regulate cardiac inflammation?
- Is RhoA activity protective in diabetic cardiomyopathy and sepsis-induced cardiac dysfunction?

## Introduction

Heart failure is a clinical syndrome characterized by structural or functional impairment of ventricular filling or ejection of blood resulting in insufficient perfusion to meet metabolic demands and is often the end-stage manifestation of cardiovascular diseases. Despite extensive research, heart failure remains a leading cause of morbidity and mortality globally [1]. There are two main etiologies of heart failure, ischemic and non-ischemic cardiomyopathy. Coronary artery disease is the most common type of heart disease, causing impaired blood flow to the heart and irreversible ischemic damage (myocardial infarction: MI) [1]. Re-establishing blood flow is critical to salvage the ischemic myocardium, but paradoxically induces and exacerbates tissue injury (ischemia/reperfusion injury: I/R injury) [1]. Nonischemic heart failure is not a well-defined diagnostic entity and includes all causes of decreased heart function other than those caused by blockages in the coronary arteries. Long-lasting hypertension can eventually lead to heart failure and it is established that diabetes is associated with heart failure (diabetic cardiomyopathy) [1]. Although aging is not a disease, it is a significant risk factor for heart failure: The prevalence of heart failure in the young adult population is less than 1%, but this rises to 8.4% after the age of 75 [2].

Rat sarcoma virus (Ras) homolog family member A (RhoA) is a member of the Ras superfamily of small GTPases that plays a central role in a range of biological processes. RhoA was discovered by Madaule and Axel as a first Ras homolog in 1985 [3] and subsequent studies established the paradigm that RhoA functions as a molecular switch for actin reorganization regulating cell shape, migration, and contraction [4–6]. Since then RhoA signaling research has expanded and it is now appreciated as a signaling hub for induction of transcriptional and post-transcriptional responses to regulate diverse cellular functions including cell growth, proliferation and survival [7–11]. Extensive studies have been devoted to understanding the role of RhoA signaling pathway in the heart and it has been increasingly clear that RhoA and its downstream effectors play significant roles in the heart. In this review, we summarize regulation of RhoA signaling pathways in the heart and discuss their roles in cardiac pathophysiology.

## RhoA signaling pathway

### Activation of RhoA

**Fig. 1:**
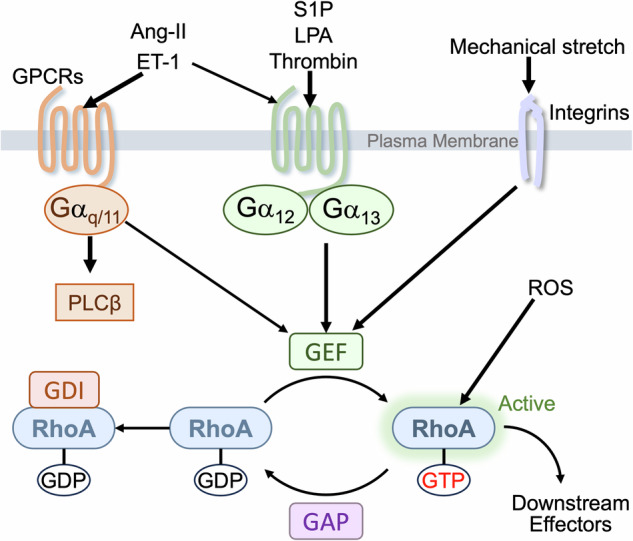
Regulation of RhoA activity by GPCR ligands and stress signal. The heterotrimeric Gα_12/13_ protein is the most established G-protein that couples receptors to RhoGEFs to activate RhoA. In some systems, Gq protein also stimulates GEFs to activate RhoA. RhoA is also activated in response to mechanical stretch and oxidative stress. Termination of RhoA activity is regulated by GAPs and RhoGDIs. GAPs accelerate the rate of hydrolysis of GTP by RhoA and GDIs inhibit the release of GDP.

Activation of RhoA is mediated by Rho guanine nucleotide exchange factors (RhoGEFs), which catalyze the exchange of GDP for GTP on RhoA, leading to RhoA activation [4–6, 12] (Fig. 1). RhoGEFs comprise approximately 80 members in humans [13, 14]. Termination of RhoA activity is regulated by GTPase activating proteins (GAPs) and Rho GDP dissociation inhibitors (RhoGDIs). RhoGAPs increase the rate of hydrolysis of GTP by RhoA leading to its inactivation and RhoGDIs inhibit the release of GDP to keep RhoA inactive [12].

#### GPCRs

The seminal article of Ridley and Hall in 1992 demonstrated that RhoA is activated in response to serum and that a major RhoA activating factor in serum is lysophosphatidic acid (LPA), a G-protein coupled receptor (GPCR) ligand [15]. It was subsequently established that ligands for GPCRs such as sphingosine-1-phosphate (S1P), thrombin, and thromboxane A2 are also efficacious activators of RhoA [7, 16–18]. In neonatal rat ventricular myocytes (NRVMs), RhoA has been shown to be activated by S1P, LPA, angiotensin-II (Ang-II), and endothelin-1 (ET-1) but not by adrenoceptor agonists (isoproterenol, phenylephrine) or muscarinic agonist (carbachol) [19–23]. In response to stimulation of GPCRs, the α subunits of heterotrimeric G-proteins (Gα_q/11_, Gα_12/13_, Gα_s_, Gα_i_) are activated and transduce their signals to downstream effectors. The mechanism by which GPCR signaling activates RhoA was first discovered by the Sternweis laboratory in 1998: The α-subunit of G_13_ (Gα_13_) binds to and activates p115-RhoGEF to elicit RhoA activation [24]. Subsequently Gα_12_ was also shown to regulate p115-RhoGEF, indicating that RhoA activity is regulated by Gα_12/13_ family proteins (Fig. 1) [25, 26]. Additional work further identified other RhoGEFs such as PDZRhoGEF, LARG and Lbc-RhoGEF regulated by Gα_12/13_ proteins [13, 14, 27]. In cardiomyocytes, we previously demonstrated that S1P-induced activation of RhoA is mediated by S1P_3_ receptor subtype-dependent Gα_13_ activation [21]. Although endothelin receptor-A (ET_A_) and angiotensin-II receptor-1 (AT_1_) are generally considered as Gα_q/11_ coupled receptors, it has been shown that inactivation of Gα_13_, but not of Gα_q/11_, abrogates RhoA activation induced by ET-1 and Ang-II in NRVMs and in adult mouse ventricular myocytes (AMVMs) [28]. A recent large-scale functional interaction study analyzed and characterized GPCRs coupling to specific heterotrimeric G-proteins and expanded the list of GPCRs that couple to Gα_12/13_ proteins such as prostaglandin F receptor and prostaglandin E2 receptor 3 [29]. These GPCRs would thus be regulators of RhoA in the heart. Although phospholipase Cβ (PLCβ) is the principal effector of Gα_q_ signaling, there is evidence that, in some systems, RhoGEFs (p63RhoGEF and TRIO) can be activated by Gα_q_ [30, 31]. The role of these RhoGEFs in cardiomyocytes has not, however, been well established.

#### Mechanical stress

RhoA signaling represents a major mechanosensitive pathway (Fig.1). Mechanical force applied to cell adhesion receptors (e.g. integrins or cadherins) activates RhoA through activation of RhoGEFs (e.g. LARG, p115RhoGEF and GEF-H1) and inhibition of RhoGAPs (e.g. DLC1 and p190RhoGAP), converting mechanical stretch into biochemical signals [32, 33]. RhoA is activated in NRVMs in response to stretch [23, 34]. Mechanical stress due to hemodynamic overload, one of primary triggers of cardiac remodeling, activates RhoA in the heart, in which integrin β1-mediated activation of LARG has been suggested to play an essential role [27, 28, 35].

#### Oxidative stress

RhoA is also activated in response to reactive oxygen species (ROS) through oxidation of RhoA [36–38]. Direct activation of RhoA by ROS requires two critical cysteine residues, Cys16 and Cys20 [36–38]. We have previously shown that RhoA is activated by hydrogen peroxide in cardiomyocytes and suggested that this could be involved in RhoA activation induced by I/R observed in the perfused heart [10, 39, 40].

### RhoA effectors

**Fig. 2:**
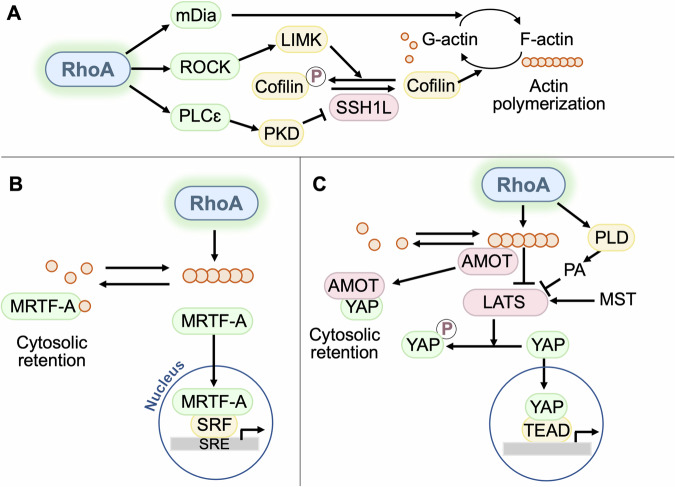
Downstream effectors of RhoA in the heart. **A** RhoA regulates actin polymerization through ROCK, mDia and PLCε. ROCK phosphorylates and activates LIMK, leading to phosphorylation and inactivation of the actin-severing protein cofilin. mDia accelerates actin nucleation while interacting with actin filament fast-growing ends. PLCε is activated by RhoA and leads to PKD activation. PKD, in turn, phosphorylates and inhibits SSH1L, a cofilin phosphatase. **B** MRTF-A associates with G-actin and is thus sequestrated in the cytoplasm under resting conditions. Activation of RhoA induces polymerization of G-actin to form F-actin filaments, freeing MRTF-A to translocate to the nucleus. **C** YAP is activated by RhoA through actin polymerization-dependent inhibition of LATS. YAP inhibition is also mediated by PA produced by PLD and by AMOT binding to YAP. RhoA-induced F-actin accumulation displaces AMOT from YAP and promotes YAP nuclear entry.

As a downstream of many GPCRs and stress signals, RhoA activation is involved in regulation of diverse biological processes that are mediated by multiple effectors of RhoA. We discuss here RhoA effectors that have been suggested to regulate cardiac pathophysiology (Fig. 2).

#### Rho-associated coiled-coil-containing kinase (ROCK)

Earlier work on RhoA signaling focused on how activation of RhoA controls actin cytoskeleton to regulate cell shape, migration and contraction and identified ROCK as the first downstream effector of RhoA [41–43]. ROCK is a serine/threonine kinase and binding of active RhoA to the Rho-binding domain in ROCK leads to its activation. There are two isoforms of ROCK, ROCK1 and 2, that share overall 65% homology at the amino acid level [44–46]. ROCK1 is ubiquitously expressed, whereas ROCK2 is enriched in some tissues such as colon, eyes, kidney, brain, and heart [44, 47]. Among the best studied and physiologically important contractile targets of ROCK is the myosin phosphatase target subunit 1 (MYPT1). MYPT1 is one of the regulatory subunits of myosin-light-chain phosphatase (MLCP) that dephosphorylates the myosin light chain (MLC) [48]. The functional role of MYPT1 in cardiomyocytes has not been known since MYPT1 is most abundantly expressed in smooth muscle cells which are also a component of the heart [49]. ROCK phosphorylates and inhibits MYPT1 which leads to increase in phosphorylation of myosin light chain and thereby increases calcium sensitivity of the contractile elements of smooth muscle [50, 51]. ROCK stabilizes actin filaments through LIM kinases (LIMK) activation. LIMK phosphorylates cofilin and inhibits its actin depolymerization activity (Fig. 2A) [52]. Y-27632 is the first small molecule ROCK inhibitor and shown to lower blood pressure in a rat model of hypertension [51]. Fasudil (HA-1077) was originally reported to inhibit PKA and PKC [53], but later found to be more potent for inhibiting ROCK [54].

#### Mammalian homolog of diaphanous (mDia)

mDia was identified as a Rho effector that induces actin filaments upon activation in 1997 [55]. Besides its Rho binding domain, mDia has two formin homology domains and belongs to the formin family and there are three mDia isoforms in mammals, mDia1, mDia2 and mDia3 [5, 56]. mDia accelerates actin nucleation while interacting with actin filament fast-growing ends, inducing actin polymerization (Fig. 2A). mDia cooperatively works with ROCK to regulate the formation of actin stress fiber [5, 57].

#### Phospholipase Cε (PLCε) and protein kinase D (PKD)

PLCε was discovered in Caenorhabditis elegans [58] and the human form of PLCε was cloned in 2001 [59]. PLCε is the only isoform of PLC that has a GTP-RhoA binding insertion and that acts as a direct RhoA effector [60–63]. In addition, PLCε contains other regulatory domains, two Ras association homology (RA) domains and a CDC25 homology GEF domain that are not found in other PLC isoforms [64]. Because of its regulation by small G-proteins, unlike the other PLCs, PLCε mediates sustained signaling. Specifically, it has been suggested that PLCβ predominantly accounts for acute and PLCε for sustained agonist-stimulated phosphatidylinositol (PI) hydrolysis [63, 65, 66]. Sustained generation of diacylglycerol and activation of its regulated kinases PKC mediates sustained activation of PKD [63, 66]. PKD phosphorylates and inhibits Slingshot 1 L (SSH1L), a cofilin phosphatase, leading to increase in phosphorylated and inactivated cofilin (Fig. 2A) [10, 39].

#### Myocardin-related transcription factor-A (MRTF-A)

RhoA activation mediated by GPCR agonists regulates gene transcription. An earlier study demonstrated that LPA activates serum response factor (SRF) through RhoA activation and that this is not mediated by the previously described transcriptional coactivator, ternary complex factor (TCF) [67]. The transcriptional coactivator downstream of RhoA was identified as a member of the myocardin family of proteins, MRTF-A (also known as MAL or MKL) [68, 69]. MRTF-A associates with G-actin and is thus sequestrated in the cytoplasm under resting conditions (Fig. 2B). Activation of RhoA induces polymerization of G-actin to form F-actin filaments, freeing MRTF-A to translocate to the nucleus, and this triggers activation of SRF target genes that are involved in a variety of cellular processes such as migration, fibrosis, proliferation and differentiation [68–70].

#### Yes-associated-protein (YAP)

YAP was first discovered as a tyrosine kinase Yes-associated protein in chicken in 1994 and the human and mouse homologs were identified in 1995 [71, 72]. YAP is a transcriptional coactivator and plays an important role in the regulation of organ size, proliferation, and cell survival [73–76]. The mammalian Hippo pathway composes core kinases, MST1/2 (macrophage stimulating 1 and 2) and LATS1/2 (large tumor suppressor kinase 1 and 2), inhibiting YAP. MST1/2 phosphorylate and activate LATS1/2, which in turn, phosphorylate and inhibit YAP. (Fig. 2C) [73–76]. When the Hippo pathway is turned off, dephosphorylated YAP translocates into the nucleus and primarily binds to TEAD, a transcription factor, to regulate expression of a wide range of genes [73–76]. YAP is activated by diverse cellular signals such as mechanical stress, extracellular matrix stiffness and GPCR signaling [8, 77–79]. LPA, S1P and thrombin activate YAP through Gα_12/13_ and RhoA signaling, and this is shown to be mediated by actin polymerization-dependent LATS1/2 inhibition [8, 77]. A recent study further demonstrated that phospholipase-D (PLD) activation and resultant phosphatidic acid (PA) production are involved in RhoA-mediated LATS1/2 inhibition and YAP activation [80]. In addition to the inhibition of LATS1/2, RhoA-mediated actin polymerization results in sequestration of angiomotin (AMOT), an inhibitory binding protein of YAP, thereby promoting YAP nuclear translocation [81, 82].

## RhoA in the heart

### Basal effect of RhoA in the heart

An earlier study using transgenic mice expressing constitutively active RhoA (L63RhoA) under the control of the cardiac-specific α-myosin heavy chain promoter revealed that cardiac expression of L63RhoA from birth results in the development of dilated cardiomyopathy and bradycardia [83]. Overexpression of L63RhoA in NRVMs for 48 h induces apoptosis through regulation of BAX, an apoptotic BCL2 family protein [84], suggesting that supraphysiological and prolonged activation of RhoA is deleterious in the heart. On the contrary, tetracycline-off inducible expression of L63RhoA (2-5 fold) in the adult heart (from the age of 11 weeks) does not alter cardiac structure, dimensions and contractility nor induce cardiac remodeling such as hypertrophy and fibrosis [10]. Notably, this study also demonstrated that chronic expression of active RhoA in the heart beginning in early development induces cardiac hypertrophy at the age of 4 months and suggests that the induction of hypertrophy in response to RhoA activation reflects signaling events that occur during heart development but not in adulthood [10]. Furthermore, cardiac-specific RhoA KO (RhoA^fl/fl^ x αMHC-Cre; RhoA cKO) mice are born at the expected Mendelian ratio and do not have an overt phenotype [9]. Specifically, cardiac structure, size, and contractility are similar in RhoA cKO and control mice [9]. Thus the basal level of RhoA activity is not indispensable in the heart, and physiological levels of activation of RhoA in the adult mouse heart is not inherently pathological. Instead, accumulating evidence reveals that the modest activation of RhoA is protective rather than maladaptive in the heart under stress conditions, as discussed below.

### RhoA and pressure-overload induced heart failure

**Fig. 3:**
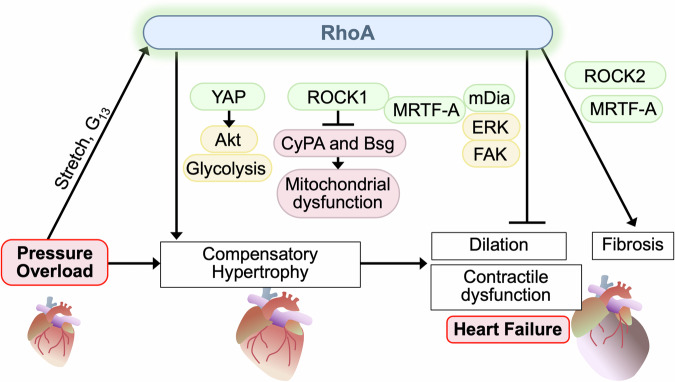
RhoA in cardiomyocytes provides protection against pressure overload by regulation of compensatory hypertrophy and inhibition of cardiac dysfunction but enhances fibrosis. RhoA is activated in response to pressure overload and contributes to the development of compensatory hypertrophy and protects the heart against cardiac dilation and contractile dysfunction. The protective effects of RhoA are mediated by YAP, ROCK1, and MRTF-A-mDia pathways. However, RhoA facilitates cardiac fibrosis possibly through activation of ROCK2 and MRTF-A.

Cardiac hypertrophy is considered to be an initial adaptive response to hemodynamic stress and necessary for normalizing ventricular wall stress and maintaining cardiac output. However, when stress is sustained, the heart transitions from compensated hypertrophy to dilated heart failure. Transverse aortic constriction (TAC) is widely used to study pressure overload-induced heart failure in mice. RhoA is activated in the heart in response to TAC through stretch-dependent and/or Gα_13_-mediated signaling mechanisms [27, 28, 35]. Since the discovery of ROCK inhibitors, many studies have focused on examining the role of ROCK in the heart and suggested that ROCK contributes to the development of heart failure induced by pressure overload [85–91], as discussed in detail later, and this lead to the hypothesis that activation of RhoA/ROCK pathway is maladaptive in the heart. Surprisingly, a study using RhoA cKO mice, however, demonstrated that cardiac deletion of RhoA leads to greater dilation, with thinner ventricular walls and larger chamber dimensions, and aggravates contractile dysfunction induced by TAC, suggesting that RhoA is required for compensatory hypertrophy and important in maintaining cardiac dimensions and contractility against TAC (Fig. 3) [9]. The salutary effects are associated with activation of cardioprotective kinases such as Akt and ERK [9]. However, cardiac deletion of RhoA inhibits cardiac fibrosis induced by TAC, indicating the contribution of cardiomyocyte RhoA to the development of fibrosis in the heart [9]. These results suggest that cardiac hypertrophy and fibrosis are independently regulated processes and that it is important to delineate the downstream effectors of RhoA in the heart.

YAP activity is increased in the mouse heart in response to TAC as well as in human hypertrophic cardiomyopathy [35, 92] and this response has been suggested to be mediated by RhoA [35]. Cardiac-specific heterozygous YAP KO mice exhibit no basal phenotype, with respect to mouse survival, heart size, or contractile function [93], but show attenuated compensatory hypertrophy and exaggerated cardiac dysfunction induced by TAC [35, 94], demonstrating the protective role of YAP. Akt activation through downregulation of PTEN and transcriptional upregulation of aerobic glycolysis are demonstrated to be involved in YAP-mediated cardioprotection against TAC (Fig. 3) [35, 94]. Pharmacological inhibition of ROCK has been shown to attenuate cardiac hypertrophy, dilation, contractile dysfunction and fibrosis induced by pressure overload [85–91], suggesting that systemic inhibition of ROCKs (ROCK1 and ROCK2) is overall cardioprotective. However, recent seminal studies have demonstrated that ROCK1 and ROCK2 are functionally different, playing the opposing roles in cardiomyocytes. Specifically, TAC-induced hypertrophy, fibrosis, cardiac dilation and contractile dysfunction are augmented in cardiac-specific ROCK1 KO (ROCK1 cKO) mice, whereas these responses are attenuated in ROCK2 cKO mice [95, 96]. Decreased hypertrophy and fibrosis were also observed in ROCK2 cKO mice subjected to Ang-II infusion [96]. These results clearly indicate that ROCK1 protects but ROCK2 jeopardizes heart failure induced by pressure overload and that ROCK1 in cardiomyocytes could contribute to the cardioprotective effects of RhoA. Mechanistically, ROCK1 exerts anti-oxidative effects through inhibition of expression of cyclophilin A (CyPA) and basigin (Bsg), both of which augment oxidative stress [95]. Given that ROCK1 and ROCK2 share 65% homology in their amino acid sequence and 92% homology in their kinase domain [45], it is intriguing that ROCK1 and ROCK2 can play such opposite roles in the heart. Earlier studies in non-cardiomyocytes reported the distinct functions of ROCK isoforms [97, 98]. For instance, a study using mouse embryonic fibroblasts (MEFs) derived from ROCK1 KO and ROCK2 KO mice demonstrates that phosphorylation of myosin light chain 2 is mediated by ROCK1 and ROCK2, while phosphorylation of cofilin is regulated mainly by ROCK2 [97]. Differential substrate specificity of ROCK isoforms in the heart has, however, not been determined. The activation kinetics of the isoforms in response to stress have not also been analyzed in the heart. Thus further studies using genetically modified mouse lines will be required to understand the underlying mechanisms of the functional differences of ROCK1 and ROCK2 in the stressed heart.

A recent study suggested that mDia1 is also involved in the development of compensatory hypertrophy in response to pressure overload through mechanotransduction-dependent activation of ERK, focal adhesion kinase (FAK), and MRTF-A, preventing the progression to heart failure [56]. MRTF-A is, however, also suggested to be responsible for enhanced fibrosis by RhoA in the TAC heart [9, 88]. Thus MRTF-A activation appears to play both protective and deleterious roles in the heart under pressure-overload stress. It is worth to note that ROCK regulates diverse cellular functions in many types of cells, not only cardiac pathophysiology, but also contractility of vascular smooth muscle cells, endothelial barrier function, myofibroblast differentiation and regulation of immune cell differentiation and function [5, 99, 100]. This might be, at least in part, responsible for conflicting data on the effect of global vs. cardiac-specific inhibition of ROCK on cardiac pathophysiology.

Together, these studies suggest that RhoA activation in cardiomyocytes plays a significant role in the development of compensatory hypertrophy induced by pressure overload, maintaining cardiac dimension and contractility. YAP, ROCK1, and mDia1 in cardiomyocytes are likely to contribute to the RhoA-mediated salutary effects (Fig. 3). Activation of RhoA in cardiomyocytes, however, increases fibrotic responses induced by TAC, which might be regulated by ROCK2/MRTF-A pathway. Further studies will be required to fully determine the involvement of these molecules in RhoA-mediated responses in the TAC heart. It would also be of importance to examine the role of RhoA in other pressure-overload models (e.g., angiotensin-II infusion model), using genetically modified mouse lines.

### RhoA and Ischemic Injury

Adult cardiomyocytes are highly differentiated cells and their regenerative capacity is limited thus cell death plays a critical role in the development of heart failure induced by ischemic stress [101–103]. Mitochondria are the main site of ATP generation in cardiomyocytes, but in response to stress, mitochondria become damaged and play a major role in cell death. Mitochondrial apoptosis is induced by release of apoptotic factors such as cytochrome *c* from mitochondria and regulated by apoptotic BCL-2 family proteins, such as BAK and BAX. This process is counteracted by anti-apoptotic BCL-2 proteins, such as BCL-2 and BCL-xL, but enhanced by BH3-only proteins such as BAD and t-BID [101–103]. The critical event in the induction of mitochondrial necrosis is the opening of the mitochondrial permeability transition pore (mPTP), a high-conductance and non-specific channel spanning both outer and inner mitochondrial membranes. mPTP is activated by mitochondrial Ca^2+^ overload and increased ROS levels, leading to dissipation of the mitochondrial membrane potential, swelling and eventual rupture of the mitochondrial membranes [101–103].

**Fig. 4:**
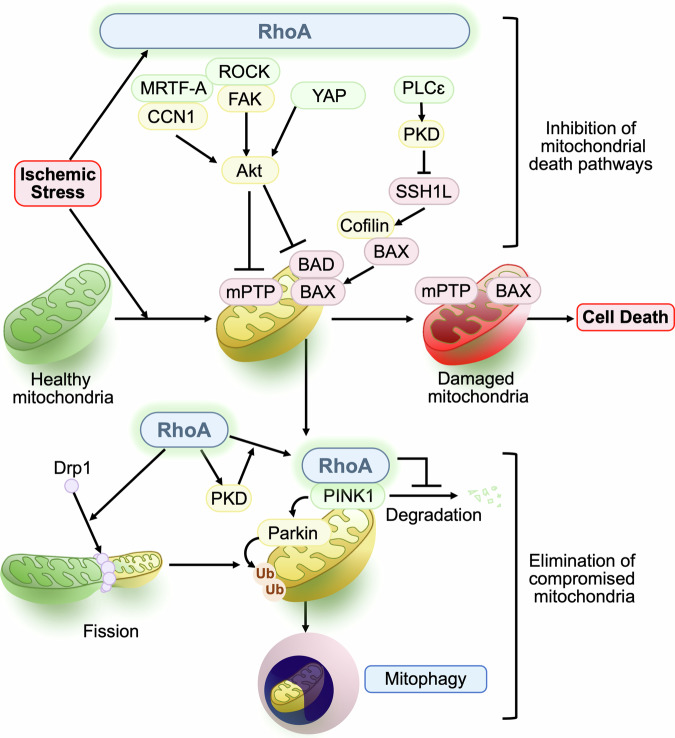
RhoA in cardiomyocytes preserves mitochondrial integrity and provides protection against ischemic stress. RhoA is activated by ischemic stress and engages multiple downstream molecules such as YAP, ROCK, MRTF-A and PLCε to activate protective kinase Akt, ERK and PKD, leading to inhibition of mitochondrial death pathways. RhoA also increases mitochondrial fission through regulation of Drp1 and enhances mitophagy through inhibition of PINK1 protein degradation to control mitochondrial quality in the ischemic heart.

RhoA is activated in response to ischemic stress in the heart [10, 40, 104] and this response could occur through activation of GPCRs in response to released mediators and/or as a direct result of oxidative stress. Studies in NRVMs demonstrated that modest levels of RhoA activation inhibit cell death induced by oxidative stress [23, 105]. Importantly, conditional, and cardiac-specific L63RhoA TG mice show reduced infarct size after I/R in vivo, while cardiac-specific RhoA KO mice show increased I/R damage in the Langendorff heart [10]. These studies strongly suggest that RhoA activation in cardiomyocytes confers protection against I/R (Fig. 4). Activation of RhoA has also been demonstrated to contribute to the protective effects of GPCR agonists against I/R. For instance, S1P, a lysophospholipid that is generated and released at sites of tissue injury, activates RhoA to provide cardioprotection against I/R [10, 19, 21, 39, 106, 107]. Our recent work further demonstrated that AAV9-mediated expression of L63RhoA in the mouse heart reduces infarct size induced by ischemia [11], thus activation of RhoA in cardiomyocytes is protective against I/R and MI.

It has been demonstrated that RhoA engages multiple downstream effectors to regulate cell survival. Akt signaling inhibits the mPTP opening as well as BAD activation to prevent mitochondrial death pathways [103, 108–110] and there are some evidences suggesting that activation of Akt contributes to the RhoA-mediated cardioprotection. Mechanistically, ROCK-dependent activation of FAK is suggested to link RhoA signal to Akt activation [23] and ROCK-MRTF-A dependent expression of CCN1, a matricellular protein, is also suggested to lead to Akt activation through the integrin signaling pathway [19]. A study using cardiac-specific heterozygous deletion of YAP demonstrated that Akt activity is also regulated by YAP in the I/R heart, contributing to YAP-mediated protection [93]. The protective effect of YAP was supported by subsequent studies demonstrating that cardiac-specific activation of YAP ameliorates cardiac damage after I/R and MI, in which cardiomyocyte proliferation and inhibition of inflammatory responses are suggested to contribute to the YAP-mediated cardioprotection [111, 112]. Global inhibition of ROCK has been established to reduce infarct size in the I/R heart [91, 104, 113–117], suggesting the deleterious role of ROCK. As discussed above, however, the site of inhibition of ROCK signaling responsible for its salutary effects may not be the cardiomyocyte and ROCK1 and ROCK2 play the opposing roles in the heart subjected to pressure overload. It would be of importance to determine if ischemic injury is also differently regulated by ROCK1 and ROCK2. PKD is another recently identified downstream molecule involved in RhoA-mediated cardioprotection. RhoA activates PKD through PLCε, and PKD in turn phosphorylates and inhibits SSH1L, leading to inactivation of cofilin (Fig.2A). This results in prevention of cofilin-mediated active BAX translocation to mitochondria and resultant cell death (Fig. 4) [10, 39].

In addition to inhibition of mitochondrial death pathways, mitochondrial integrity is also preserved by mitochondrial quality control mechanisms. Mitochondria-specific autophagy (mitophagy) removes damaged mitochondria through lysosomal degradation, inhibiting the accumulation of toxic mitochondria and resultant cell death [118–121]. One of the best-established mechanisms of tagging damaged mitochondria for autophagic removal is the mitochondrial membrane-depolarization-dependent PTEN-induced kinase 1 (PINK1)/Parkin pathway. Dissipation of the mitochondrial membrane potential induced by stress stabilizes and accumulates PINK1 at the mitochondrial outer membrane. PINK1, in turn, recruits Parkin, an E3 ubiquitin ligase, which ubiquitinates and tags damaged mitochondria for removal [118, 119]. Although PINK1/Parkin-mediated mitophagy is generally protective, full depolarization of the mitochondrial membrane potential occurs just before cell death, and additional intracellular mechanisms that enhance the PINK1/Parkin pathway at early stages would be important for prolonging cell survival but such a mechanism has not been fully explored. We recently discovered that RhoA signaling increases mitophagy by stabilizing PINK1 protein at mitochondria without concomitantly diminishing mitochondrial membrane potential and provides cardioprotection against MI (Fig. 4) [11, 122]. Regulation of PINK1/Parkin-mediated mitophagy by RhoA was further confirmed by a recent study [123]. Mechanistically, we demonstrated that RhoA, upon its activation, translocates to mitochondria and interacts with PINK1 and that the molecular interaction of RhoA with PINK1 prevents PINK1 degradation, ensuing sequelae of events culminating in mitophagy. Interestingly, RhoA translocation to mitochondria is regulated by PKD [11, 122]. Although the precise mechanism by which RhoA prevents PINK1 degradation at mitochondria remains to be elucidated, our results suggest that receptor agonists and interventions that activate RhoA represent a novel means of stabilizing PINK1 to stimulate mitophagy without compromising mitochondrial functions to promote cell survival. Activation of RhoA-ROCK pathway leads to phosphorylation of Drp1 and its translocation to mitochondria to induce mitochondrial fission [105], a cellular process that segregates damaged mitochondria from intact mitochondrial network and facilitates autophagic removal of damaged mitochondria (Fig. 4) [124]. In addition to mitochondrial fission, recent studies have revealed that Drp1 can directly regulate ULK1-Rab9-dependent alternative mitophagy in the heart [125]. Whether RhoA regulates alternative mitophagy to protect the heart is an interesting unanswered question.

Taken together, these studies suggest that mitochondria are a convergence point for RhoA-mediated cellular protection, with RhoA signaling through multiple pathways to preserve mitochondrial integrity by preventing mitochondrial death pathways and by facilitating removal of damaged mitochondria during ischemic stress.

### RhoA and other heart diseases

**Fig. 5:**
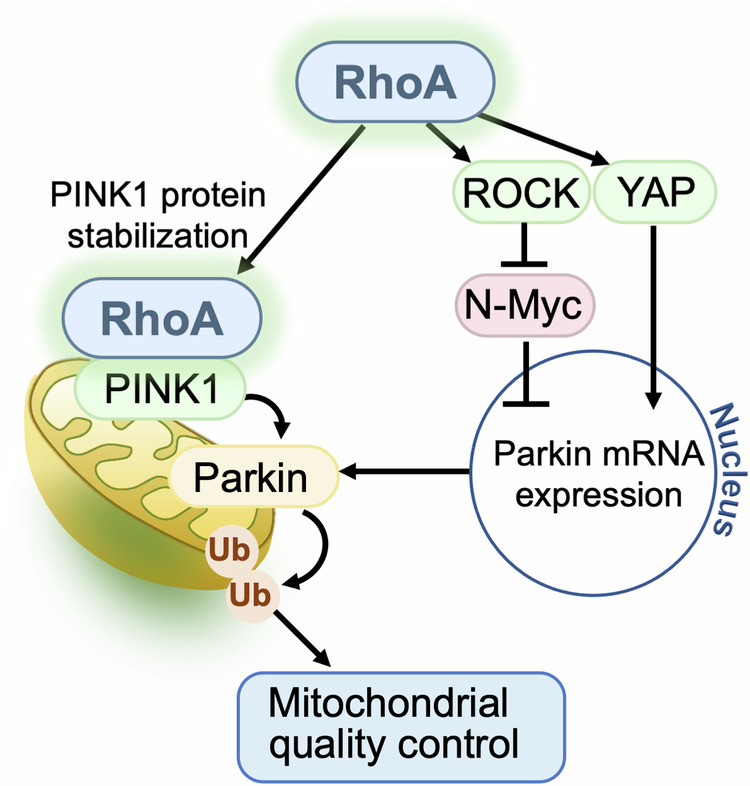
RhoA signaling regulates PINK1/Parkin-mediated mitophagy to preserve mitochondrial integrity. PINK1 protein stability is regulated by the molecular interaction of RhoA with PINK1. Parkin expression is transcriptionally regulated by ROCK through inhibition of N-Myc as well as YAP.

Aging is a significant risk factor for heart failure and results in progressive deterioration in the structure and function of the heart [2]. Dysfunctional proteins and mitochondria are accumulated in the aging heart and enhancing autophagic clearance exerts beneficial effects [126]. A recent study using RhoA cKO mice demonstrated that RhoA-mediated mitophagy provides cardioprotection against aging-induced adverse cardiac remodeling, including cardiac hypertrophy, fibrosis, dilation, and contractile dysfunction [123]. ROCK-dependent transcriptional upregulation of Parkin through inhibition of N-Myc, a transcriptional repressor of Parkin, is suggested to contribute to RhoA-mediated mitophagy in the aging heart (Fig. 5) [127]. An earlier study also noted that Parkin mRNA levels are decreased in ROCK1 cKO and in ROCK2 cKO mice [95]. YAP has also been demonstrated to regulate Parkin gene expression to protect the heart against doxorubicin-induced cardiotoxicity [128]. Thus it appears that RhoA regulates PINK1/Parkin-mediated mitophagy through early regulation of PINK1 protein stabilization [11, 122] and late transcriptional upregulation of Parkin [95, 123, 128] to ensure cell survival against acute and sustained stress.

There is some evidence that RhoA signaling can regulate general autophagy in the heart.

For instance, inducible ROCK1 and ROCK2 double cKO mice show no overt basal phenotype but decreased fibrosis by aging and this fibrotic effect of ROCK is suggested to be attributed to inhibition of autophagy [129]. However, the inhibitory effect of ROCK on autophagy was not evident when ROCK1 or ROCK2 was individually deleted from adult cardiomyocytes [129]. The role of RhoA/ROCK signaling in autophagy in the heart requires further studies.

Diabetic cardiomyopathy describes diabetes-associated changes in the structure and function of the myocardium that occur independently of a recognized cause such as hypertension and coronary artery disease. RhoA expression and activity in the heart has been demonstrated to be increased in streptozotocin-diabetic rats [130–132]. However, whether this response is adaptive or deleterious has not been determined. Pharmacological studies have shown that ROCK inhibition ameliorates diabetes-induced cardiac dysfunction [131–133] and a study using heterozygous ROCK2 KO mice demonstrated that ROCK2 phosphorylates ryanodine receptors, impairing cardiac Ca^2+^ homeostasis [134]. Inhibition of MST1, an upstream negative regulator of YAP, alleviates diabetic cardiomyopathy [135–137], implying the possibility that YAP plays a protective role in diabetic hearts. MST1, however, not only inhibits YAP but also induces apoptosis through inhibition of BCL-xL [138] and it would be of importance to further elucidate the roles of RhoA and its downstream effectors in diabetic cardiomyopathy.

Sepsis is a systemic inflammatory response triggered by bacterial infection. Cardiac dysfunction is an important component of multi-organ failure and resultant mortality induced by severe sepsis. Several studies have shown that ROCK inhibitors provide salutary effects in the heart against septic shock [130, 139, 140]. In contrast to ROCK, YAP activity has been shown to be protective against lipopolysaccharide (LPS)-induced cardiac dysfunction [141, 142]. YAP activation is also shown to suppress inflammatory responses in NRVMs treated with LPS [111] and the anti-inflammatory effects of YAP in the heart have also been reported in ischemic heart models [111, 143, 144]. On the contrary, YAP has been reported to enhance inflammatory responses in fibroblast and macrophages [145, 146].

## Conclusion

It was originally discovered that RhoA plays a major role in regulation of cytoskeletal function in smooth muscle and other cells and that ROCK is the main downstream effector of RhoA. It is now evident that RhoA can regulate diverse cellular functions and plays an important role in the heart in response to stress. In addition to ROCK, various signaling molecules such as YAP, MRTF-A and PLCε have been discovered to play an important role in RhoA-mediated transcriptional and post-transcriptional regulation. Recent evidence demonstrates that RhoA in cardiomyocytes prevents the development of heart failure induced by stress, such as pressure overload, ischemia and aging. RhoA-mediated transcriptional regulation contributes to the development of compensatory hypertrophy but also enhances fibrotic responses in the heart. The protective effect of RhoA is derived, at least in part, from preservation of mitochondrial integrity through inhibition of mitochondrial cell death pathways as well as through regulation of mitochondrial quality control.

Although our understanding of the role of RhoA signaling pathway in the heart has expanded greatly in recent years, there are unsolved questions that warrant future research. Further studies of the role of RhoA signaling pathway in regulation of mitochondrial quality control mechanisms including PINK1/Parkin independent mitophagy, mitochondrial dynamics and mitochondrial biogenesis will be required. General autophagy also plays a critical role in maintaining cardiac homeostasis. There is, however, only limited data available with regard to regulation of general autophagy by RhoA in the in vivo heart. Cardiac inflammation is evident in the failing heart [147] and now considered to be a driver of the adverse cardiac remodeling [148, 149], but the role of RhoA signaling in regulation of inflammation in the heart is still emerging and many more research will be required to determine how RhoA signaling regulates cardiac inflammation through regulation of inflammatory gene expression and through regulation of cell survival in the heart.

RhoA controls many different biological functions in different types of cells, thus a deeper understanding of the intracellular signaling molecules regulated by RhoA in cardiac cells as well as in non-cardiac cells is obligatory for identifying precise targets for modulating RhoA signaling pathway to provide a new therapeutic strategy for prevention or treatment of heart disease.
